# Mitochondria-penetrating peptides conjugated to desferrioxamine as chelators for mitochondrial labile iron

**DOI:** 10.1371/journal.pone.0171729

**Published:** 2017-02-08

**Authors:** Roxana Y. P. Alta, Hector A. Vitorino, Dibakar Goswami, Cleber W. Liria, Simon P. Wisnovsky, Shana O. Kelley, M. Terêsa Machini, Breno P. Espósito

**Affiliations:** 1 Department of Fundamental Chemistry, Institute of Chemistry, University of São Paulo, São Paulo, Brazil; 2 Department of Biochemistry, Institute of Chemistry, University of São Paulo, São Paulo, Brazil; 3 Bhabha Atomic Research Centre, Mumbai, Maharashtra, India; 4 Department of Biochemistry, University of Toronto, Toronto, Ontario, Canada; 5 Faculty of Pharmacy, University of Toronto, Toronto, Ontario, Canada; CINVESTAV-IPN, MEXICO

## Abstract

Desferrioxamine (DFO) is a bacterial siderophore with a high affinity for iron, but low cell penetration. As part of our ongoing project focused on DFO-conjugates, we synthesized, purified, characterized and studied new mtDFOs (DFO conjugated to the Mitochondria Penetrating Peptides TAT_49-57_, 1A, SS02 and SS20) using a succinic linker. These new conjugates retained their strong iron binding ability and antioxidant capacity. They were relatively non toxic to A2780 cells (IC50 40–100 μM) and had good mitochondrial localization (Rr +0.45 –+0.68) as observed when labeled with carboxy-tetramethylrhodamine (TAMRA) In general, mtDFO caused only modest levels of mitochondrial DNA (mtDNA) damage. DFO-SS02 retained the antioxidant ability of the parent peptide, shown by the inhibition of mitochondrial superoxide formation. None of the compounds displayed cell cycle arrest or enhanced apoptosis. Taken together, these results indicate that mtDFO could be promising compounds for amelioration of the disease symptoms of iron overload in mitochondria.

## Introduction

Transition metals are critical for enzyme function and protein folding, but excesses can mediate neurotoxic oxidative processes [1]. Iron is the most important transition metal for biological systems, being a cornerstone of several redox and electron transfer processes such as cellular respiration and energy production. As energy production involves oxidative phosphorylation, a process requiring a continuous flow of electrons, mitochondria are particularly vulnerable to oxidative damage [2]. As such, mitochondria are the major sites of Reactive Oxygen Species (ROS) generation, which are produced as byproducts of the electron transport chain. Since free iron and certain ROS can engage into potentially deleterious processes such as the Fenton reaction, mitochondrial iron homeostasis must be tightly controlled.

Iron overload is therefore potentially harmful to organisms due to the promotion of oxidative stress in aerobic conditions. Iron overload may be a result of inherited dysfunctions in the regulation of iron homeostasis (e.g. hereditary hemochromatosis) or of the treatment of an unrelated condition (e.g. after multiple blood transfusions for the treatment of thalassemias) [3]. Also, iron overload may be systemic or localized in specific tissues or cell organelles. Examples of the latter include neurodegenerative conditions such as Friedreich Ataxia (FA) which is related to increased concentration of mitochondrial labile iron [4].

Iron chelation is the first line of treatment for most cases of iron overload, although reaching specific targets or compartments (e.g., the inner mitochondrial space) poses additional challenges. Clinically-approved chelators for the treatment of iron overload are desferrioxamine (DFO), deferiprone and deferasirox. DFO is the only natural product and the first bacterial siderophore to be introduced for medical use. It has a strong affinity and high specificity for iron, however it is not cell-permeable due to its elevated hydrophilicity [5]. Deferiprone, on the other hand, permeates several physiological barriers and therefore shows promise for the treatment of iron overload in neurological disorders such as FA (despite being discontinued [6, 7]), Alzheimer’s Disease or Parkinson’s Disease [8]. Deferasirox has promoted modest improvement of the symptoms of FA [9].

Previous works in our groups demonstrated the effectiveness of conjugating specific peptide sequences to DFO in order to render it cell-permeable [10]. As a continuation of this effort, in the present work we studied the possibility of rendering DFO mitochondria permeable by conjugating this moiety with mitochondria-penetrating peptides (MPPs), which are highly cationic and minimally toxic peptide sequences that selectively localize to mitochondria in cells [11, 12]. We selected MPPs with both good cell permeation and mitochondrial localization, including the trans-activating transcriptional activator (TAT_49-57_; R-K-K-R-R-Q-R-R-R [13]), 1A (F_X_-r-F_X_-K-F_X_-r-F_X_-K-NH_2_ [11]) and the tetrapeptides SS02 (Dmt-r-F-K-NH_2_) and SS20 (F-r-F-K-NH_2_) [14]. *d*-Arginine (r) renders the peptides less prone to be cleaved by cytosolic proteases, while cyclohexylalanine (F_X_) is highly lipophilic, endowing the peptide with the hydrophobicity necessary to traverse the highly hydrophobic mitochondrial inner membrane. 2’,6’-dimethyltyrosine (Dmt) in SS02 is an antioxidant and, therefore, an extra protection against free radical damage; SS20 is its control.

**Fig 1:**
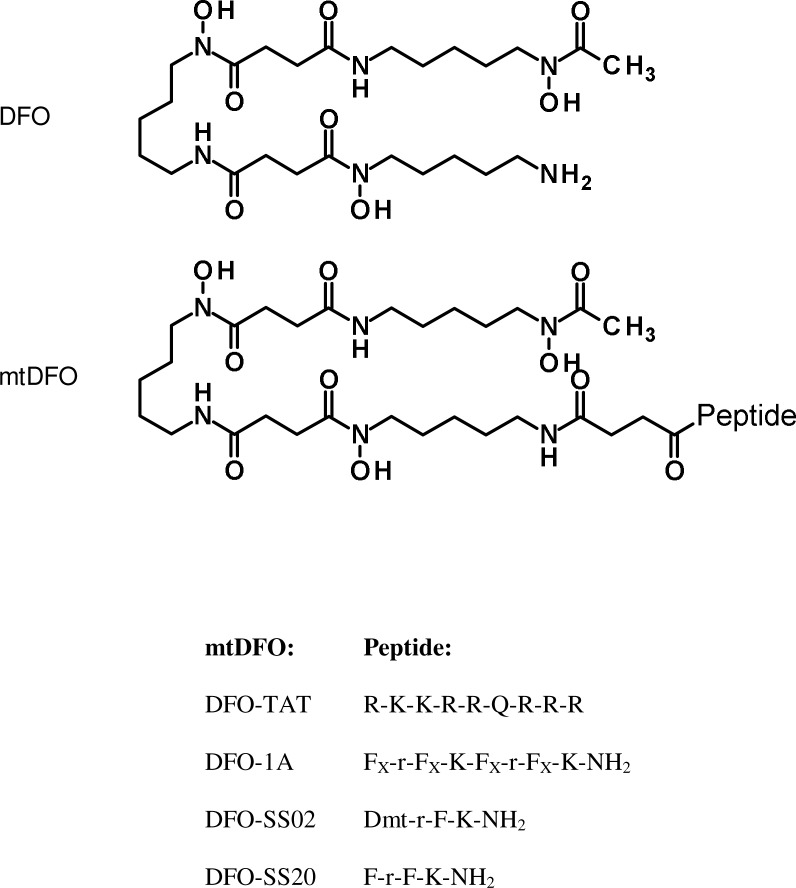
Structures of DFO and mtDFO in this study. The succinyl linker is grayed. Dmt: 2’,6’-dimethyltyrosine; F_X_: cyclohexylalanine; r: *d*-arginine.

Here we report that the selected MPPs were synthesized and linked to DFO by means of succinylation following the synthetic strategy established in our previous studies [10, 15], although most steps were carried out at 60°C using conventional heating. After purification and characterization, the four mitochondria-targeted DFO conjugates (mtDFO, Fig 1) had their iron binding and antioxidant (against an iron-dependent oxidation model) properties studied. mtDFO were also synthesized in their carboxy-tetramethylrhodamine (TAMRA)-labeled form to demonstrate proper mitochondrial localization in A2780 cells. Also, mtDFO displayed low levels of toxicity, cell cycle arrest, mtDNA damage and apoptosis. DFO-SS02 significantly quenched ROS generation in mitochondria.

## Materials and methods

### Materials

Fmoc-Arg(Pmc)-OH, Fmoc-Arg(Pbf)-OH and Fmoc-Gln-OH from Novabiochem (USA); Fmoc-Lys(Boc)-OH, Fmoc-β-cyclohexyl-Ala-OH, Fmoc-D-Arg(Pmc)-OH and Fmoc-Phe-OH from Bachem (USA); Fmoc-L-(2,6-di-Me)Tyr-OH from AnaSpec (Belgium); Rink-Amide-(Fmoc)-resin (0.7 mmol/g) from Advanced ChemTech (USA); Fmoc-Arg-(Pmc)-Wang resin (0.61 mmol/g) from Novabiochem (USA); 1,3-Diisopropylcarbodiimide (DIC) from Advanced ChemTech (USA); N,N,N′,N′-Tetramethyl-O-(1H-benzotriazol-1-yl)uronium hexafluorophosphate (HBTU) from ChemPep Inc. (USA); 1-Hydroxybenzotriazole (HOBt) from Bachem (USA); N,N-diisopropylethylamine (DIPEA) and ninhydrin from Applied Biosystems (USA). Peptides were synthesized manually or on a Prelude Protein Technologies peptide synthesizer as described previously [16]. DFO mesylate (Desferal) was donated by Novartis (USA) or Cristália (Brazil). Piperidine, triisopropylsilane, trifluoroacetic acid (TFA), thioanisole, 1,2-ethanedithiol, 4-(2-Hydroxyethyl)piperazine-1-ethanesulfonic acid (HEPES), nitrilotriacetic acid (NTA), ferrous ammonium sulfate hexahydrate (FAS), ascorbic acid, calcein, bovine apo-transferrin and 5-(4,6-dichlorotriazinyl)aminofluorescein (5-DTAF) were obtained from Sigma-Aldrich (USA). Succinic anhydride was purchased from Vetec Fine Chemicals Ltd. (Brazil). FeCl_3_ and phenol were purchased from LabSynth (Brazil). Dihydrorhodamine (DHR) was obtained from Biotium (USA). Deferiprone was donated by Apotex (Canada). All reagents were of analytical grade and used as received without further purification.

The solvents dichloromethane (DCM) and methanol (analytical grade) were purchased from Merck (Germany), whereas dimethyl sulfoxide (analytical grade) was obtained from Sigma-Aldrich (USA). Dimethyl sulfoxide (DMF) (analytical grade) and acetonitrile (ACN) (chromatographic grade) were purchased from Vetec Fine Chemicals Ltd. (Brazil). HBS (Hepes Buffered Saline; NaCl 150 mM, HEPES 20 mM; pH 7.4; treated with Chelex-100 purchased from Sigma, 1 g/ 100 mL) was used throughout the experiments.

### Synthesis, purification and chemical characterization of MPP and mtDFO

The manual syntheses starting from variable amounts of Fmoc-Arg(Pmc)-Wang resin (for TAT_49-57_) or Rink-Amide-(Fmoc) resin (for 1A, SS-02 and SS-20) were performed at room temperature or 60°C following the synthetic strategy and protocols described in our previous works [10, 17], although most steps were performed at 60°C using conventional heating. Succinylation followed by coupling of DFO was performed according to our previous work [17]. MPP or mtDFO cleavage from resin and simultaneous full deprotection were achieved by treating the peptide-resins with appropriate mixtures of TFA and scavengers at 37°C to be described elsewhere. The peptides or mtDFO were precipitated from the reaction medium with cold diethyl ether, separated by centrifugation and purified by semipreparative HPLC using suitable conditions. The fractions containing the desired compounds were pooled and lyophylized. The purities of the final MPP and mtDFO were assessed via analytical HPLC and LC/ESI-MS. Their peptide contents were determined after full acidic hydrolysis followed by amino acid analysis of the hydrolyzated.

### Synthesis, purification and chemical characterization of TAMRA-mtDFO

The automatic syntheses were conducted traditionally at room temperature as reported starting from Rink-Amide-MBHA resin [11, 18]. The N-deprotected peptidyl-resin was washed with DMF. Succinylation followed by coupling of DFO was performed according to our previous work [17]. A mixture of 200 mmol DFO and 200 mmol HBTU was dissolved in 1.5 mL of 10% DIPEA in DMF (v/v) and added to the N-Suc-peptidyl-resin. A 50-mmol portion of Fmoc-peptide-MPP was deprotected in a 3% solution of TFA in DCM for 30 min and coupled to (5,6)-TAMRA using four equivalents (5,6)-TAMRA, four equivalents HBTU, and eight equivalents DIPEA in DMF for 2 h. The resulting resin was labeled with 200 mmol of DFO using the procedure described above for the preparation of mtDFO. The dry resin was treated with a specific cocktail reagent. mtDFO was precipitated from the cleavage solution with diethyl ether. The resulting solid mtDFO was separated by centrifugation and purified by semipreparative HPLC. Fractions from sequential runs containing mtDFO were pooled and lyophilized. The purity of the final product was assessed via analytical HPLC. Identification was done by MALDI-TOF.

### Competition studies with the calcein-iron (CAFe) complex

Calcein-iron (CAFe) is a complex which is rendered fluorescent by the scavenging activity of other chelators (releasing in solution the fluorescent molecule calcein), such as DFO. CAFe (10 mM in calcein and in iron) is prepared by dissolving 3.92 mg of FAS in 1.00 mL of calcein 10 mM. Aliquots of 190 μL of CAFe (2 μM in HBS, pH 7.4) were placed in flat, transparent 96-well microplates. They were treated with increasing concentrations of DFO, MPP and mtDFO under screening (10 μL aliquots) and allowed to react at 37°C for 1 h. The fluorescence was recorded on a BMG FluoStar Optima instrument (λ_exc_/λ_em_ = 485/520 nm) [19].

### Competition studies with fluorescein-apotransferrin (Fl-aTf)

Fl-aTf was prepared by a previously described method [20]. Mixtures of 180 μL Fl-aTf (2 μM in HBS loaded with 10 mM HCO_3_^-^) and 20 μL aliquots of chelators (DFO and mtDFO) with increasing concentrations (0−20 μM) were prepared in a 96-well microplate, and FAS (10 μL, 4 μM final concentration) was added. The reactions were allowed to proceed for 1 h at 37°C, then the fluorescence was recorded on a BMG FluoStar Optima instrument (λ_exc_/λ_em_ = 485/520 nm).

### Antioxidant activity

The standard ferric nitrilotriacetate (Fe(NTA)) complex (1:3 Fe:NTA molar ratio) was prepared by adding FAS to a stock solution of aqueous NTA, and allowed to react for 1 h at 37°C under air so that iron is oxidized to Fe(III) (evidenced by the change of color to deep yellow). Then, 180 μL of a mixture of 40 μM ascorbic acid, 50 μM DHR, and 20 μM chelators (DFO, DFO-Tat, DFO-1A, DFO-SS02 e DFO-SS20) in HBS was placed in a 96-well microplate and treated with 20 μL of either Fe(NTA) at different concentrations or sera from iron overloaded patients. Assays were performed in duplicate. Sera samples were furnished by Dr. Nelson Hamerschlak, Albert Einstein Hospital, São Paulo. Fluorescence was measured in a BMG FluoStar Optima instrument for 60 min at 37°C (λ_exc_/λ_em_ = 485/520 nm). The slopes (indicating rate of oxidation, presented in fluorescence units per minute) of the oxidation curves were calculated in the time range 15−40 min and were plotted against iron concentration [21].

### Cell culture

A2780 (human ovarian cancer) cell line was obtained from Fox Chase Cancer Center (USA) and cultured in RPMI-1640 medium supplemented with 10% FBS and 1% penicillin/streptomycin. Cells were incubated at 37°C in a humidified incubator with a 5% CO_2_.

### Cytotoxicity assays

A2780 cells were plated in 96-well flat-bottom tissue culture plates (Starstedt) at 25,000 cells per well. The culture medium was removed, and cells were washed. Cellular viability was analyzed after an overnight incubation at 37°C with 5% CO_2_ using the CCK-8 viability dye (Dojindo, Rockville, Maryland) at an absorbance of 450 nm. From the resulting dose-response curves, 50% growth inhibitory concentration (IC_50_) values were determined by interpolation [22].

### Mitochondrial localization studies

A2780 cells were seeded in an imaging dish in 2 mL of growth medium at 60% confluence. The growth medium was swapped with premixed medium containing 1–10 μM of TAMRA-mtDFO, and the cells were allowed to incubate with the dye for 1 h. MitoTracker Deep Red was added to the cells at 1.0–4.0 μM concentrations and allowed to incubate for 30 min. At the end of the incubation period, the medium was aspirated, and the cells were washed with 3 × 1 mL PBS and incubated with 2 mL of dye-free DMEM. The imaging experiments were performed using a Zeiss Axiovert 200M inverted epifluorescence microscope equipped with an EM-CCD digital camera (Hamamatsu) and a MS200 XY Piezo Z stage (Applied Scientific Instruments). The light source was an X-Cite 120 metal-halide lamp (EXFO), and the fluorescence images were obtained with an oil-immersion objective at 63× magnification. The microscope was operated by the Volocity software program of Perkin-Elmer. Colocalization of the dyes was quantitated using the program ImageJ using a previously described protocols [18, 23].

### mtDNA Polymerase Chain Reaction (PCR)

A2780 cells were seeded at 2 × 10^5^ cells/well and allowed to adhere overnight. Cells were treated with mtDFO at half of their IC_50_ for 6 h and harvested after trypsinization. DNA was isolated from cell pellets using the Sigma GenElute mammalian genomic DNA miniprep kit according to the manufacturer’s instructions. Amplification of an 8.9 kb segment of mitochondrial DNA was performed using the Elongase long range PCR enzyme kit (Invitrogen) as described previously [24]. Quantitation of amplified product was performed by Pico Green staining and normalized to non-treated value.

### Mitochondrial superoxide levels

A2780 cells were plated in 12-well plates at a density of 10^5^ cells/mL and allowed to attach overnight. Cells were then treated with half their IC_50_ concentrations of mtDFO and incubated for 24 h. The medium was removed and cells were washed with Hank’s buffered saline and then incubated with 5 μM MitoSox reagent (Invitrogen) in HBS for 30 min in the absence of light. Cells were washed three times with Hank’s buffered saline, harvested by trypsinization, and analyzed via flow cytometry with FACSCanto. At least 10^4^ cells were analyzed for each sample [18].

### Cell cycle analysis

A2780 cells were seeded in a 12-well dish at a density of 10^5^ cells/mL and allowed to adhere overnight. Cells were treated with half their IC_50_ concentrations of mtDFO and incubated for 24 h. Cells were harvested by trypsinization and fixed at 4°C in 70% ethanol for 2 h. Cells were centrifuged, ethanol was removed, and the remaining cell pellet was washed once with PBS. Cells were resuspended in PBS and incubated with 0.2 mg/mL RNase A for 1 h at 37°C, then stained with 10 mg/mL propidium iodide and analyzed immediately via flow cytometry with FACSCanto. Quantitation of data was performed using FlowJo. At least 10^4^ cells were analyzed for each sample [18].

### Annexin V apoptosis assay

A2780 cells were plated in a 12-well dish at a density of 10^5^ cells/mL and allowed to adhere overnight. Cells were treated with half their IC_50_ concentrations of mtDFO for 24 h. Following incubation, cells were stained at room temperature with Annexin V-FITC (Invitrogen kit) for 15 min and 5 nM Sytox Red for an additional 15 min. Analysis was performed via flow cytometry with FACSCanto. At least 10^4^ cells were analyzed for each sample [18].

## Results

### Manual synthesis, purification and characterization of MPP and mtDFO

The synthetic strategy previously established by our groups [10, 15, 17] was shown to be effective in the synthesis of mtDFOs. Most steps were significantly accelerated by the use of high temperature and conventional heating, not routinely used in most laboratories worldwide. The final products presented purity percentages higher than 95%. Amino acid analysis (S1 Table in Supporting Information) indicated peptide content in the range 45–82%. Manually synthesized MPP and mtDFO were used for all the tests, except for colocalization studies.

### Automatic synthesis, purification and characterization of TAMRA-mtDFO

The fluorophore-labeled analogs, TAMRA-mtDFOs, were obtained by attaching TAMRA to the amino side chain of a lysine residue added to the C-terminal of each peptide sequence. The purified TAMRA-mtDFO were characterized by MALDI-TOF (S1 Fig in Supporting Information), and these conjugates were used in the colocalization studies.

### Fluorimetric competition studies (CAFe and Fl-aTf)

**Fig 2:**
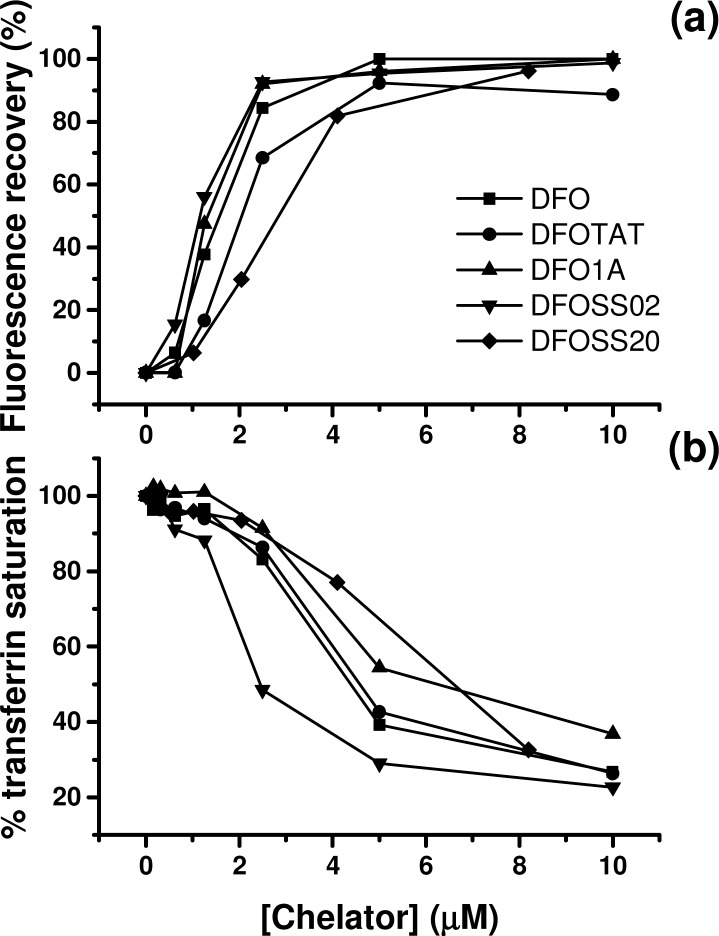
Iron-binding ability of mtDFO. (a) Fluorescence recovery promoted by mtDFO competing with 2 μM CAFe. (b) Percentage of transferrin saturation by iron when 2 μM Fl-aTf is mixed with increasing amounts of mtDFO. Results are the average of quadruplicates and representative of at least two isolated experiments. The samples do not show statistic differences.

The profiles of calcein de-quenching by both free DFO and mtDFO are very similar (Fig 2A). In the other hand, the free peptides lacking a high affinity binding moiety for iron were not able to remove iron from the CAFe complex (data not shown). When a mixture of Fl-aTf and DFO/mtDFO was treated with an iron salt, similar profiles of Fl-aTf quenching were observed (Fig 2B).

### Antioxidant activity

**Fig 3:**
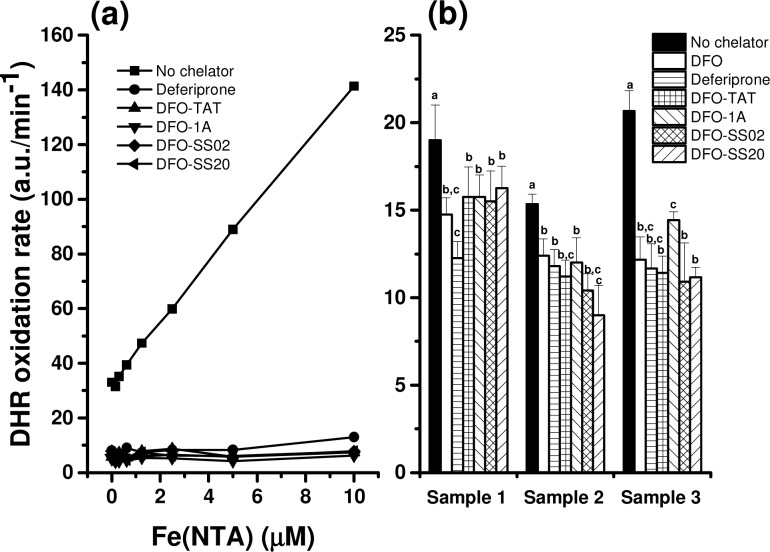
Antioxidant activity of mtDFO (20 μM). Their effects on the oxidation rate of the probe DHR treated with (a) increasing amounts of Fe(NTA) or (b) serum of iron-overloaded myelodysplastic patients are displayed. For n = 4, p < 0.05. a.u. = arbitrary units of fluorescence. Results are the average of quadruplicates and representative of at least two isolated experiments. Different letters indicate significant differences with control (no chelator) after one way ANOVA (P < 0.05).

All siderophores (free DFO and mtDFO) efficiently suppressed the iron-catalysed oxidation of the DHR probe, both in buffered medium and in samples of iron-overloaded patients (Fig 3). Free peptides were also tested but had no antioxidant effect within this concentration range (data not shown).

### Cytotoxicity assays

Half maximal inhibitory concentration (IC_50_) values for DFO, mtDFO and TAMRA-tagged mtDFO are displayed in Table 1. In general, the toxicity of mtDFO was considerably higher than that of DFO. mtDFO can be divided into two classes of toxicity (DFO-TAT ~ DFO-1A < DFO-SS02 ~ DFO-SS20). Tagging of TAMRA fluorescent marker further increases toxicity, with all TAMRA-mtDFO displaying similar IC_50_ (~ 2–8 μM).

**Table 1:** IC_50_ (μM) of mtDFO, DFO and TAMRA-tagged mtDFO in A2780 cells after 24 h.

Compounds	IC_50_
DFO	1452±130
DFO-TAT	110.80±1.17
DFO-1A	108.00±2.51
DFO-SS20	62.37±1.13
DFO-SS02	42.88±1.06
DFO-TAT-TAMRA	4.21±1.30
DFO-1A-TAMRA	2.97±1.06
DFO-SS20-TAMRA	7.91±1.19
DFO-SS02-TAMRA	2.45±1.43

### Mitochondrial localization studies

**Fig 4:**
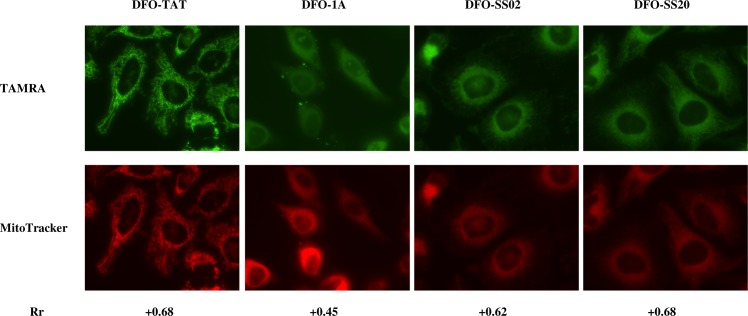
Colocalization of mtDFO(TAMRA) in mitochondria of A2780 cells. Rr = Pearson correlation coefficient.

The TAMRA probe does not locate by itself in the mitochondria. When tagged to mtDFO, fluorescence microcopy images show a high degree of co-localization with MitoTracker (Fig 4). Pearson correlation coefficients for all the TAMRA-mtDFO were high (+0.45 –+0.68), even for the TAT derivative.

### mtDNA PCR

**Fig 5:**
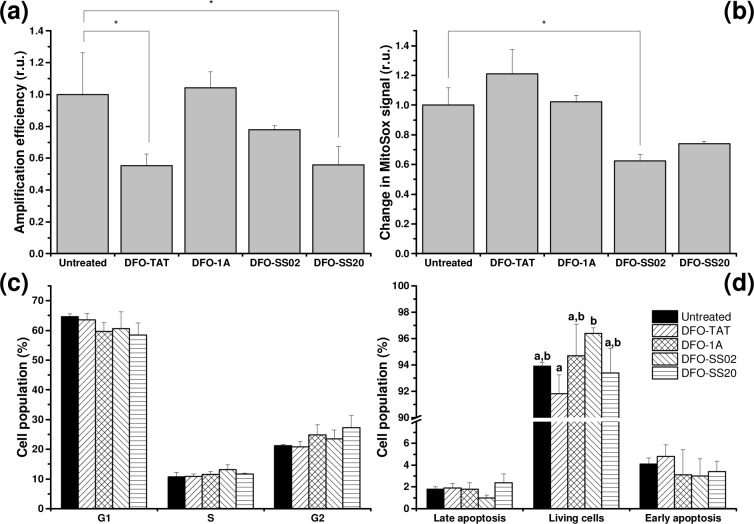
Cellular effects on A2780 cells treated with ½ IC_50_ of the mtDFO. (a) mtDNA amplification. (b) Production of mitochondrial superoxide. (c) Cell cycle arrest. (d) Apoptosis tested by the Annexin V method. r.u. = relative units; *: statistically significant differences in relation to untreated controls (P < 0.01). Different letters indicate significant differences in relation to untreated controls after one way ANOVA (P < 0.05).

A2780 cells treated with mtDFO at ½ IC_50_ had decreased efficiency of mtDNA amplification for all but DFO-1A treatment (Fig 5A).

### Mitochondrial superoxide levels

Mitochondrial ROS production in A2780 cells treated with mtDFO at ½ IC_50_ was assessed by the MitoSox probe (Fig 5B). Only the SS02 conjugate displayed significant reduction in superoxide levels.

### Cell cycle analysis

A2780 cells treated with mtDFO at ½ IC_50_ did not display significant differences among cell stages as compared to the control (Fig 5C).

### Annexin V apoptosis assay

A2780 cells treated with mtDFO at ½ IC_50_ were highly viable irrespective of the treatment (> 90%; Fig 5D). Accordingly, no significant deviations from the control were found for both late and early apoptotic populations.

## Discussion

Iron overload is a concern in a number of clinical conditions, as excess iron within the body facilitates pro-oxidant reactions with oxygen or nitrogen substrates, giving rise to oxidative stress [25]. Therefore, excess iron must be removed by means of chelation therapy with natural or synthetic siderophores (desferrioxamine, deferiprone, deferasirox). DFO is the main siderophore for clinical use. However, because of its hydrophilicity, it is not absorbable by the gastrointestinal tract and must be infused in long, daily sessions [5]. Diseases such as hereditary hemochromatosis or thalassemia are characterized by non-localized deposits of excess iron, however a number of conditions are known in which iron overload is circumscribed to a specific tissue or organelle (neurodegeneration with brain iron accumulation, hereditary X-linked sideroblastic anemia, anemia of chronic disease [26]). Patients of FA typically have a decreased expression of the peptide frataxin, which assists the assembly of iron-sulfur clusters within the mitochondria.

Mitochondria-Penetrating Peptides are short amino acid sequences permeable to both plasma and mitochondrial membranes that are comprised of lipophilic (F, Fx) and cationic (K, R) residues. MPPs have low toxicity to human cells [2, 12], and the artificial r- and Fx-based sequences are not metabolized by endogenous proteases [23]. Therefore, MPPs are interesting carrier molecules to deliver specific cargoes to mitochondria [2, 12]. In this work, we prepared four new conjugates of MPP with DFO using a succinic linker (termed “mtDFO”) as a means to have a strong iron chelator permeable to mitochondria. To the best of our knowledge, this is the first time that a clinically approved siderophore is targeted to mitochondria by means of MPP. The preservation of iron binding ability, antioxidant capacity, mitochondrial localization and lack of toxicity to A2780 cell model were also demonstrated.

As in our previous works, the synthetic strategy used by us included preparation of the MPP on a solid phase, functionalization of MPP-resin with succinic anhydride and acylation of DFO by the Suc-MPP-resin (Fig 1) using elevated temperature [10, 15, 17]. Details will be reported elsewhere. The preservation of the full iron binding ability of the mtDFO was demonstrated by experiments with two fluorescent probes. In the first one, the on/off fluorescence of calcein mediated by iron(III) binding was explored. Calcein is a fluorescent chelator, and its fluorescence is stoichiometrically quenched when bound to iron to form the complex CAFe. DFO has a higher affinity for iron than calcein, therefore DFO is able to scavenge iron from CAFe which thus restitutes the fluorescence of the system. Fig 2A shows that the iron binding and affinity of the four mtDFO were identical to that of free DFO (maximum recover at 1:1 Fe:chelator mol ratio). Also, all high affinity iron binding is due to the siderophore and not to the MPP amide backbone, as the free peptides do not display activity.

A second, and more practical probe was Fl-aTf, initially proposed by Breuer and Cabantchik as a probe of plasmatic labile iron pools of iron-overloaded patients [20]. Fl-aTf, like calcein, is a quenchable probe for iron, but the stoichiometry of binding is 2:1 (iron:transferrin), and not 1:1 (iron:calcein). Despite the fact that the affinity of DFO for iron is higher than that of transferrin, the removal of iron from Fe_2_Tf by DFO is kinetically hindered as iron is buried in a binding pocket within the protein with little exposure to the solvent [27]. Therefore, the setup of this experiment was modified accordingly. Here, Fl-aTf and varying amounts of mtDFO were mixed together, and then exogenous iron was added and left to redistribute with the mixture of ligands (Fig 2B). Again, no distinguishable behavior of free DFO and mtDFO were observed. Increasing concentrations of DFO or mtDFO led to lower saturation of Fl-aTf until a 20% baseline was reached.

It has been proposed previously that the iron-catalyzed auto-oxidation of ascorbate under physiological conditions was a clinically relevant parameter for assessing the fraction of iron most probable to cause oxidative damage in iron-overloaded patients [21]. DHR is a probe that is oxidized by the products of such catalysis, with the rate of oxidation directly dependent on the iron concentration. Clinically relevant chelators must halt this oxidation. Chelators such as DFO or deferiprone are known to have this ability in a dose-dependent manner. In the absence of a chelator, increasing concentrations of Fe(NTA) (the model of labile iron) speed the oxidation, however all mtDFOs have the antioxidant effect similar to deferiprone (a standard chelator; Fig 3A). The same beneficial trend was observed in the plasma of iron-overloaded patients (Fig 3B).

Mitochondrial localization of mtDFOs was assessed in the A2780 cell line, which has a well-defined mitochondrial network [18, 23]. The organelles were marked with MitoTracker, and the degree of co-localization of mtDFOs derivatized with TAMRA (TAMRA-mtDFO) was expressed by the Pearson correlation coefficient (Rr). With data derived from the images in Fig 4, all mtDFOs showed good Rr values ranging from +0.45 to +0.68, meaning that they all entered mitochondria. Some authors claim that TAT is not permeable to isolated mitochondria [28], however our results are in agreement with other successful cargo loading to the mitochondria facilitated by this peptide in whole tissues [29–31]. Localization in the mitochondria depends on both the number of positive charges and the lipophilicity of the peptide (or the peptide-cargo system). We found that even a relatively heavy and highly hydrophilic cargo such as DFO did not impede MPP targeting.

As it is highly hydrophilic and thus cell impermeable, the cytotoxicity of free DFO is relatively low compared with the mtDFO (Table 1; full dose-response curves are displayed in S2 Fig in Supporting Information) [32, 33]. In the other hand, the fact that DFO is only absorbed intravenously hampers its use for the treatment of patients and a loss of opportunity to access iron in specific tissues or organelles [34, 35]. The higher toxicity of mtDFO is probably because, as a strong chelator, intracellular DFO may disturb the homeostasis of any loosely bound metal ion, such as Zn(II) (logK_Zn(DFO)_ = 10.36) [36, 37]. The IC50 values are, however, still relatively high within the 40–100 μM range. In a previous work we observed that the conjugation of DFO to cell-penetrating peptides (such as penetratin) were not toxic at concentrations up to 20 μM [10, 15]. Tagging TAMRA to the mtDFO causes a further increase in toxicity due probably to the generation of singlet oxygen under laser irradiation of the probe [38].

We also studied other cellular events affected by the mitochondrial targeting of DFO. The inhibitory effect of xenobiotics on the progression of PCR of a fragment of mtDNA is a means to assess mtDNA damage [24]. After 24 h, it was observed that mtDFO with higher Rr caused the highest impact on mtDNA amplification rate, with significance being attained in two cases (Fig 5A). As DFO is a good chelator for other metal species than iron, it is possible that, to some extent, it acts as a blocker of Zn-dependent regulators of DNA transcription [27, 39]. It should be noted, however, that the concentrations of mtDFO were chosen to be half of the IC_50_ values (Table 1). Therefore, mitochondria were exposed to ~ 50 μM (DFO-Tat; DFO-1A) or 30 μM (DFO-SS02; DFO-SS20) of the DFO conjugates. These are very high concentrations which probably will not have clinical significance, meaning that even the ~50% decrease in the amplification rate would not be relevant.

Other MPP with different cargoes such as mtDOX and mtCbl were also found to induce mtDNA damage due to the cargo molecules, leading to even lower amplification rates. The levels of damage caused by mtDOX (8 μM) or mtCbl (3 μM) produced 0.9±0.1 and 1.4±0.1 lesions per 10 kb respectively [22, 40], whereas the four mtDFO produced injuries lower than 0.5 lesions per 10 kb (Fig 5A). This result indicates that the mtDFO conjugates we studied possessed minimal mtDNA damaging activity.

Approximately 1–3% of mitochondrial dioxygen is incompletely reduced, giving rise to significant amounts of superoxide, the main ROS produced by this organelle [4, 41]. Thus, we studied the effect of mtDFO on the generation of mitochondrial superoxide (Fig 5B), having found that only the conjugate of DFO with the antioxidant Dmt (DFO-SS02) induced significant protection. This confirms previous results [14] and is relevant in the sense that superoxide can oxidize Fe,S clusters and foster the release of ferrous ions and peroxide, leading to a cascade of oxidative damage [42].

Further indicating the relatively low impact of mtDFO on regular cell function, none of the conjugates induced any significant cell cycle arrest (Fig 5C) or apoptosis (Fig 5D and S3 Fig in Supporting Information). These compounds do not seem to compromise membrane integrity, even when carrying a good metal chelator.

As discussed above, we determined that DFO-SS02 has the best chemical properties related to mitochondrial localization and antioxidant activity. Even though its synthesis involves the use of an expensive amino acid (Dmt), there is demand for other mitochondrial iron chelators. Deferiprone also has access to this pool of labile iron, however its use for FA therapy was discontinued due to severe side effects such as agranulocytosis, muscular-skeletal pain, dizziness or Guillain-Barre syndrome [6, 7].

In conclusion, the production of mtDFO is an interesting strategy to load a powerful, yet impermeant siderophore into either cytoplasm or mitochondria. This could contribute to the improvement of several iron overload conditions, where iron excess is either systemic or localized into specific tissues/organelles.

## Supporting information

S1 Table Chemical analysis and characterization of mtDFO obtained by manual synthesis. (DOCX)

S1 Fig Characterization of mtDFO-TAMRA by MALDI mass spectrum. (TIF)

S2 Fig Viability of A2780 cells treated for 24 h with DFO, mtDFO or TAMRA-mtDFO. Values are the average (± s.d.) of at least three independent experiments.
(TIF)

S3 Fig Annexin V/Sytox Red staining of A2780 cells treated with mtDFO for 24 h. Percentage of cells in each quadrant is showed. a) Non treated; b) 55.4 μM DFO-Tat; c) 54.0 μM DFO-1A; d) 19.0 μM DFO-SS02; d) 21.1 μM DFO-SS20. n.v. = not viable.
(TIF)

## Abbreviations

5-DTAF: 5-(4,6-dichlorotriazinyl)aminofluorescein
ACN: Acetonitrile
CAFe: Calcein-iron
DCM: dichloromethane
DFO: Desferrioxamine
DHR: Dihydrorhodamine
DIC: 1,3-Diisopropylcarbodiimide
DIPEA: N,N-diisopropylethylamine
DMEM: Dulbecco's Modified Eagle's Medium
DMF: dimethyl formamide
Dmt: 2’,6’-dimethyltyrosine
FA: Friedreich Ataxia
FAS: ferrous ammonium sulfate hexahydrate
Fe(NTA): ferric nitrilotriacetate
Fl-aTf: fluorescein-apotransferrin
F_X_: cyclohexylalanine
HBS: Hepes Buffered Saline
HBTU: N,N,N′,N′-Tetramethyl-O-(1H-benzotriazol-1-yl)uronium hexafluorophosphate
HEPES: 4-(2-Hydroxyethyl)piperazine-1-ethanesulfonic acid
HOBt: 1-Hydroxybenzotriazole
IC_50_: half maximal inhibitory concentration
MPP: mitochondria-penetrating peptide
mtDFO: DFO conjugated to Mitochondria Penetrating Peptides
mtDNA: mitochondrial DNA
NTA: nitrilotriacetic acid
PCR: Polymerase Chain Reaction
r: d-Arginine
ROS: Reactive Oxygen Species
TAMRA: carboxy-tetramethylrhodamine
Tf: transferrin
TFA: Trifluoroacetic acid
