# Supplementary material for: Mitochondria-penetrating peptides conjugated to desferrioxamine as chelators for mitochondrial labile iron
Source: PLoS One. 2017 Feb 8;12(2):e0171729. doi: 10.1371/journal.pone.0171729 (PMC5298241; doi:10.1371/journal.pone.0171729)
Supplement: S1 Table — (DOCX) [file pone.0171729.s001.docx]

**S1 Table – Chemical analysis and characterization of mtDFO obtained by manual synthesis**

| **mtDFO** | **Molecular**  **mass (g/mol)** | | | | **Amino acids** | **Amino acid molar ratio (theoretical/found)** | **Peptide content (%)** |
| --- | --- | --- | --- | --- | --- | --- | --- |
|  | **theoretical** | **found** | **charge** | **detected ion** |  |  |  |
| DFO-TAT | 1981.5 | 1981.5  1981.5 | 4+  3+ | 496.6  661.5 | Arg  Lys  Gln | 6/5.5  2/1.7  1/1.0 | 45 |
| DFO-1A | 1841.7 | 1841.2  1841.4 | 4+  3+ | 461.3  614.8 | *d*-Arg  Lys  Fx | 2/2.0  2/2.0  4/N.D. | 46 |
| DFO-SS02 | 1282.4 | 1282.0  1281.9 | 2+  3+ | 642.0  428.3 | *d*-Arg  Lys  Phe  Dmt | 1/  1/  1/  1/N.D. | 36 |
| DFO-SS20 | 1237.7 | 1237.8  1237.8 | 2+  1+ | 619.9  1238.8 | *d*-Arg  Lys  Phe | 1/  1/  2/ | 82 |

Fx = cyclohexylalanine; Dmt = 2’,6’-dimethyltyrosine; (N.D. = not determined)
