# Supplementary figures and images for: Mitochondria-penetrating peptides conjugated to desferrioxamine as chelators for mitochondrial labile iron
Source: PLoS One. 2017 Feb 8;12(2):e0171729. doi: 10.1371/journal.pone.0171729 (PMC5298241; doi:10.1371/journal.pone.0171729)

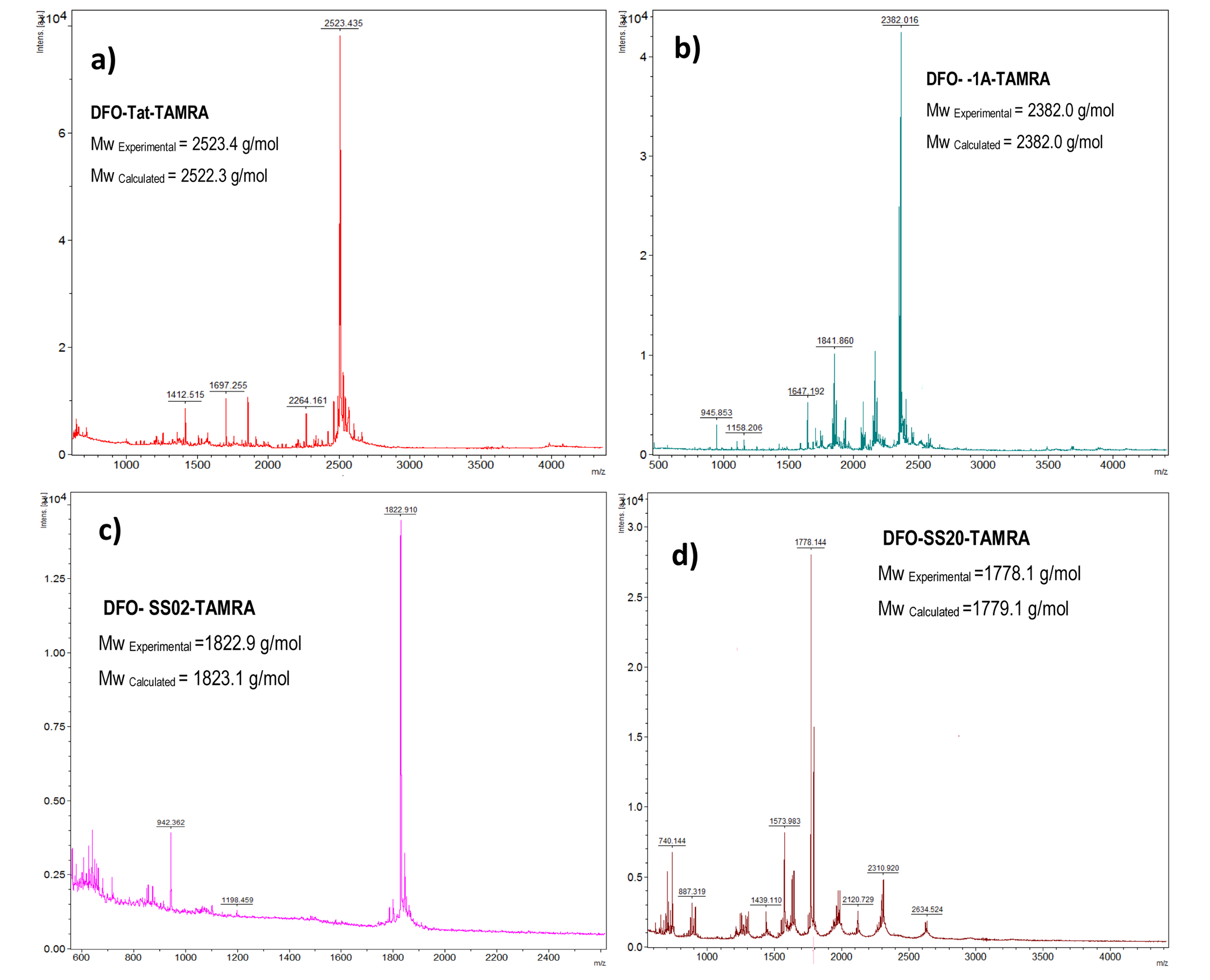

Supplement: S1 Fig — (TIF) [file pone.0171729.s002.tif]

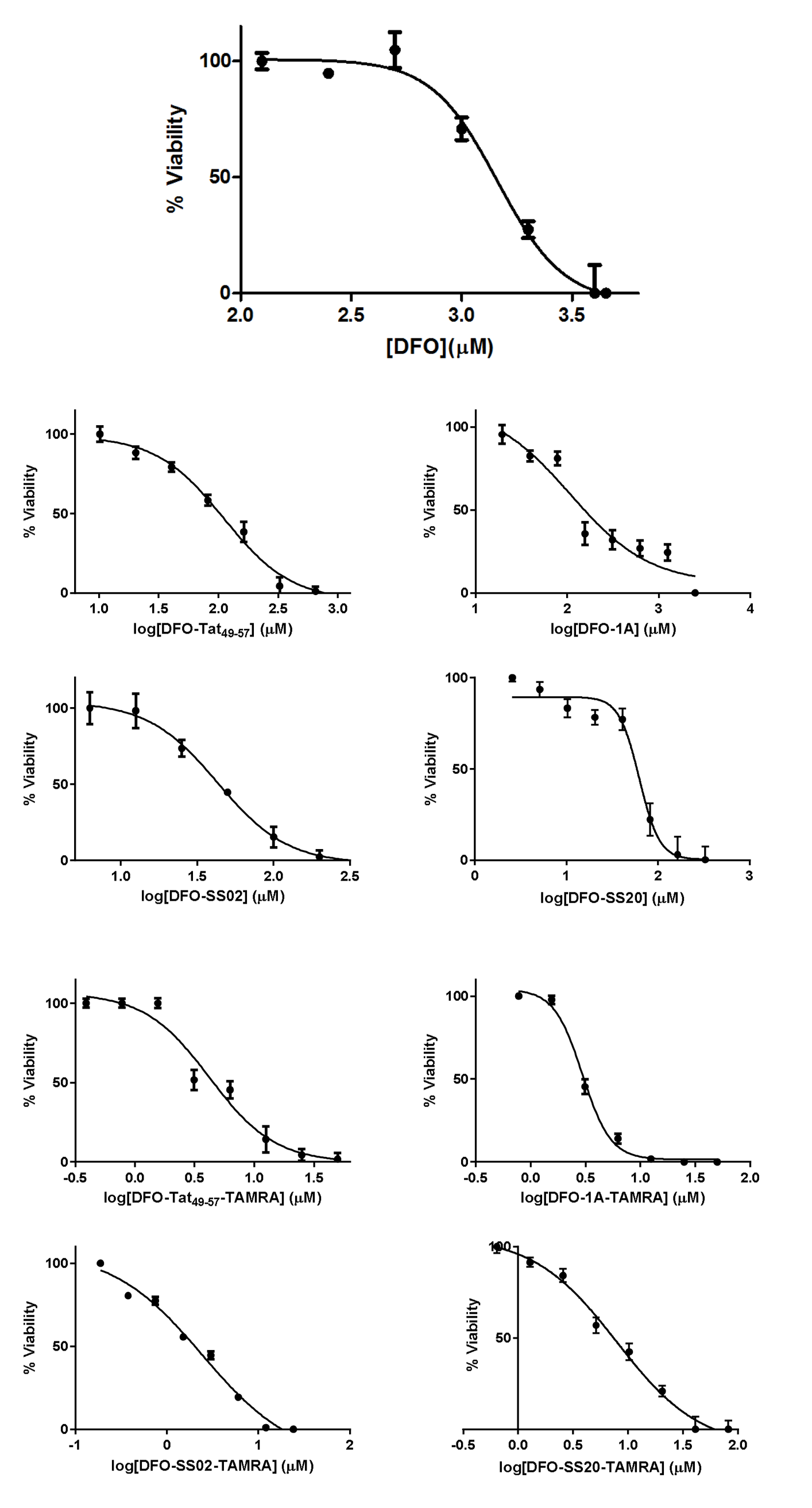

Supplement: S2 Fig — Values are the average (± s.d.) of at least three independent experiments. (TIF) [file pone.0171729.s003.tif]

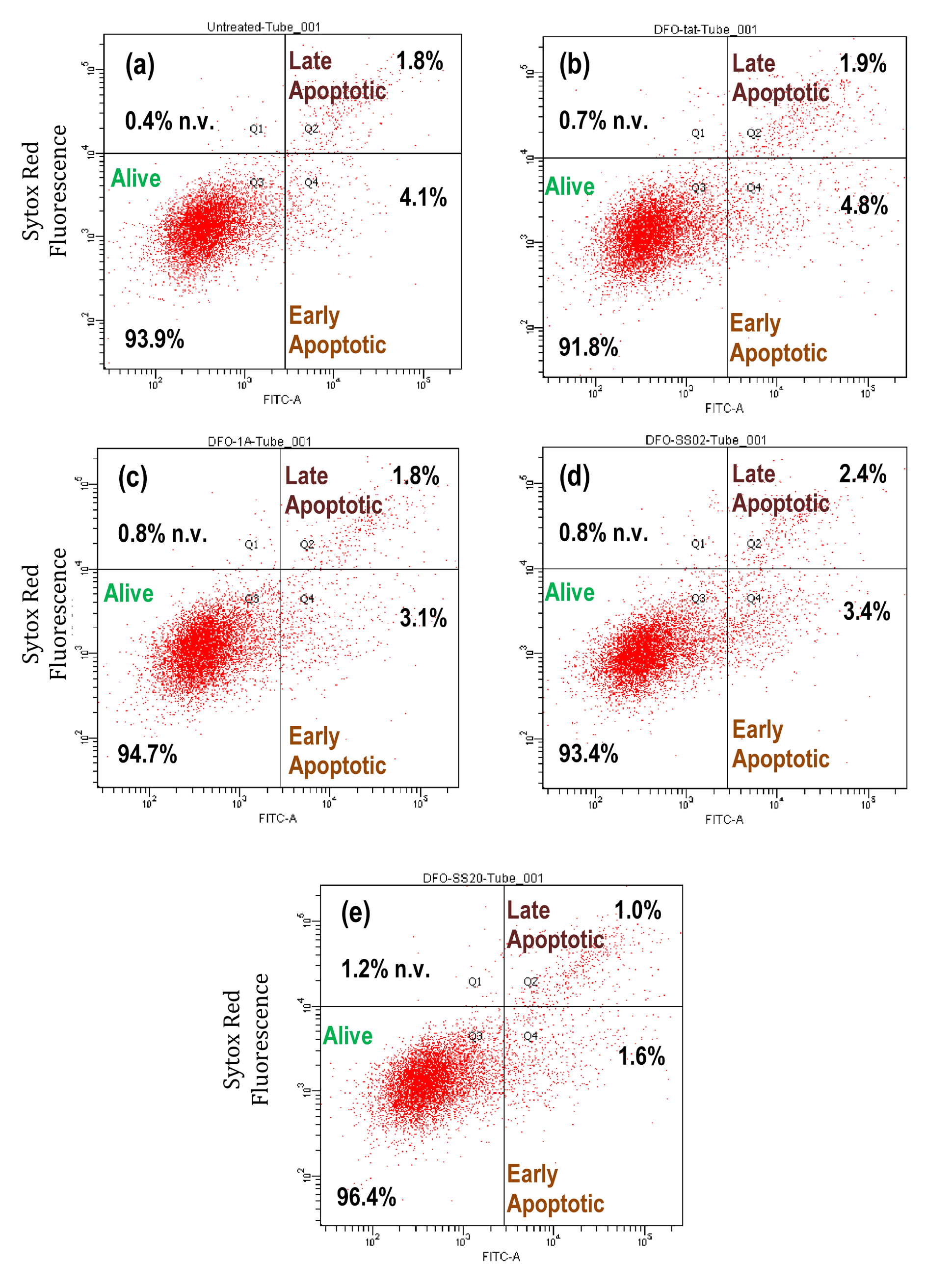

Supplement: S3 Fig — Percentage of cells in each quadrant is showed. a) Non treated; b) 55.4 μM DFO-Tat; c) 54.0 μM DFO-1A; d) 19.0 μM DFO-SS02; d) 21.1 μM DFO-SS20. n.v. = not viable. (TIF) [file pone.0171729.s004.tif]
